# Automated diagnostic support system with deep learning algorithms for distinction of Philadelphia chromosome-negative myeloproliferative neoplasms using peripheral blood specimen

**DOI:** 10.1038/s41598-021-82826-9

**Published:** 2021-02-09

**Authors:** Konobu Kimura, Tomohiko Ai, Yuki Horiuchi, Akihiko Matsuzaki, Kumiko Nishibe, Setsuko Marutani, Kaori Saito, Kimiko Kaniyu, Ikki Takehara, Kinya Uchihashi, Akimichi Ohsaka, Yoko Tabe

**Affiliations:** 1 Department of Next Generation Hematology Laboratory Medicine, Juntendo University Graduate School of Medicine, 2-1-1, Hongo, Bunkyo-ku, Tokyo, 113-8421 Japan; 2 Department of Clinical Laboratory Medicine, Juntendo University Graduate School of Medicine, Tokyo, Japan; 3 Sysmex, Kobe, Japan

**Keywords:** Myeloproliferative disease, Computational biology and bioinformatics

## Abstract

Philadelphia chromosome-negative myeloproliferative neoplasms (Ph-negative MPNs) such as polycythemia vera (PV), essential thrombocythemia (ET), and primary myelofibrosis are characterized by abnormal proliferation of mature bone marrow cell lineages. Since various non-hematologic disorders can also cause leukocytosis, thrombocytosis and polycythemia, the detection of abnormal peripheral blood cells is essential for the diagnostic screening of Ph-negative MPNs. We sought to develop an automated diagnostic support system of Ph-negative MPNs. Our strategy was to combine the complete blood cell count and research parameters obtained by an automated hematology analyzer (Sysmex XN-9000) with morphological parameters that were extracted using a convolutional neural network deep learning system equipped with an Extreme Gradient Boosting (XGBoost)-based decision-making algorithm. The developed system showed promising performance in the differentiation of PV, ET, and MF with high accuracy when compared with those of the human diagnoses, namely: > 90% sensitivity and > 90% specificity. The calculated area under the curve of the ROC curves were 0.990, 0.967, and 0.974 for PV, ET, MF, respectively. This study is a step toward establishing a universal automated diagnostic system for all types of hematology disorders.

## Introduction

Philadelphia chromosome–negative myeloproliferative neoplasms (Ph-negative MPNs) are a group of hematological disorders that result from malignant transformations of hematopoietic stem cells^1,2^, and are characterized by abnormal proliferation of mature bone marrow (BM) cell lineages (i.e., granulocytes, erythrocytes, and megakaryocytes), which include polycythemia vera (PV), essential thrombocythemia (ET) and primary myelofibrosis (PMF)^3^.

In Ph-negative MPNs, genetic variants in *JAK2*, *CALR*, and *MPL* are instrumental in activating downstream pathways that drive excessive myeloproliferation such as STAT, MAPK/ERK, and PI3/AKT^4^. Genetic tests help identify these variants, which are useful to determine the disease type of Ph-negative MPNs. However, these results alone cannot define the types of Ph-negative MPNs; this is because the gene variants overlap with Ph-negative MPNs. Therefore, WHO emphasizes the importance of an accurate evaluation of the morphological features of BM^5^. Additionally, the diagnoses of MPNs further require a careful evaluation of the clinical history and past physical examinations of the patient. Further, a histology of their BM and affected organs is necessitated along with their molecular genetic data.

As the initial diagnostic workup, complete blood cell counts (CBCs) and peripheral blood (PB) smears are essential. Since various non-hematologic disorders can also cause leukocytosis, thrombocytosis, and polycythemia, careful evaluation of the morphology in PB cells is critical for accurate initial diagnoses of Ph-negative MPNs, especially for detecting abnormalities in the cells. For example, immature granulocytes and nucleated RBCs are known to be observed in MF, including overt PMFs and secondary MFs^6,7^.

In hematological laboratories, large amounts of blood samples are sent without clinical information. Although automated hematology analyzers can evaluate CBCs and cell types to some extent, lab technologists are often required to examine PB smears with the microscopes, which is very tedious. In order to reduce the workload and inter- and intra-personal inconsistency, we previously developed an automated image analysis system using deep convolutional neural networks (CNNs) based-image analysis algorithms using a total of 695,030 normal and abnormal blood cells. Using the system, we could differentiate myelodysplastic syndrome (MDS) and aplastic anemia with high accuracy compared to human diagnoses^8^.

In this study, we sought to further develop an automated diagnostic support system for Ph-negative MPNs by combining an automated hematology analyzer (Sysmex XN-9000) and the previously built CNN-based deep learning system (DLS)^9^. We trained this new combined system with image and CBC data obtained from MPN samples, then evaluated the feasibility and accuracy of the system to differentiate PV, ET and MF.

## Results

### Use of morphological parameters to differentiate Ph-negative MPNs

**Figure 1:**
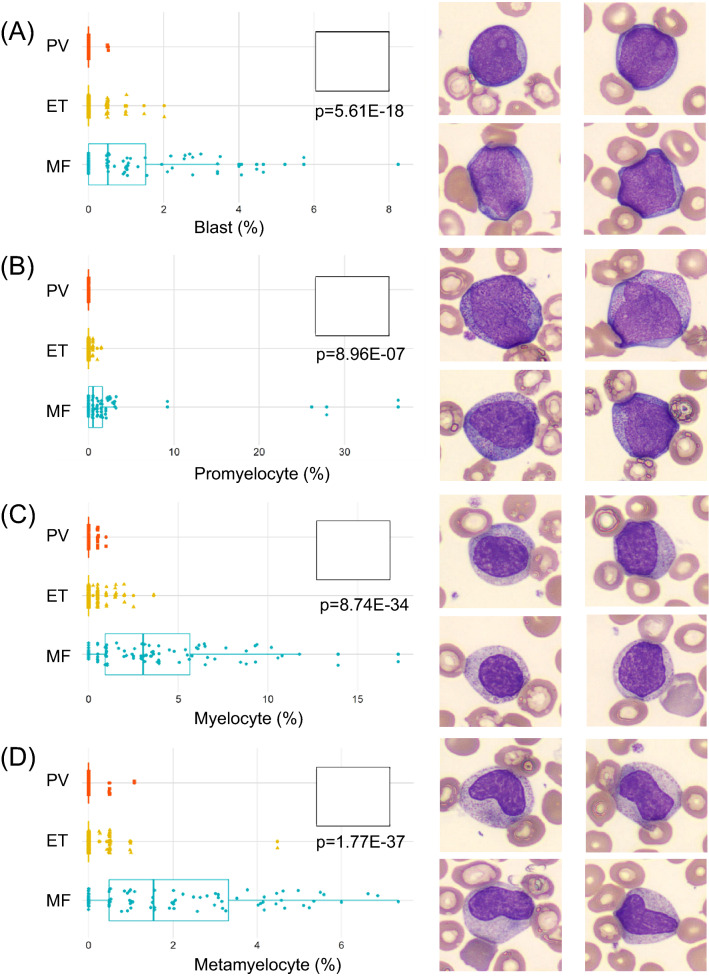

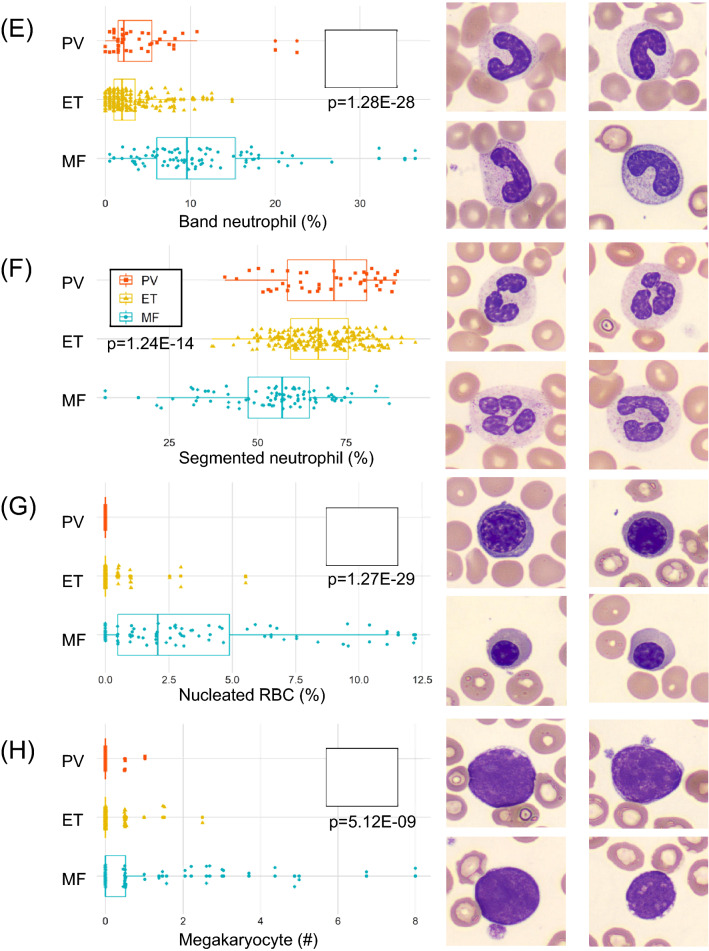

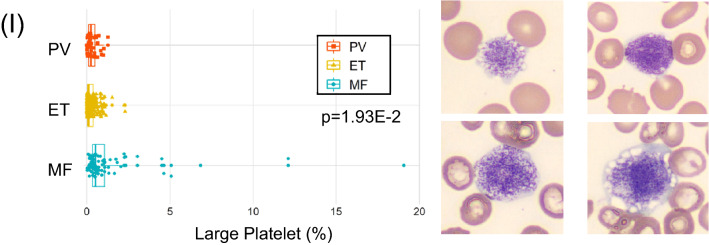
CNN-based morphological DLS parameters in differentiating Ph-negative MPNs. (**A**-**I**) The box plots (left panels) represent the percentage of cell counts for every 200 leukocytes classified by the DLS for each case of PV, ET, and MF. The right-hand side panels show representative cell images for each cell category.

Figure 1 outlines the useful morphological DLS parameters for PV, ET, and MF. The box plots (left panels) represent percent cell counts for every 200 leukocytes classified by the CNN-based image analysis per case. The panels to the right show representative cell images for each cell category. Blasts and immature granulocytes, such as promyelocyte, myelocyte, and metamyelocyte, were observed to appear more in MF than in PV and ET (Fig. 1A-D). In MF, the ratio of the band neutrophils over the total leukocytes increased, and the ratio of the segmented neutrophils decreased (Fig. 1E,F). Nucleated RBCs also appeared more in MF than in PV and ET (Fig. 1G). Similarly, megakaryocytes and giant platelets appeared more in MF than PV or ET (Fig. 1H,I). In contrast, no unique morphological features were observed in the comparison between PV and ET (data not shown).

### Use of XN-9000 parameters to differentiate Ph-negative MPNs

**Figure 2:**
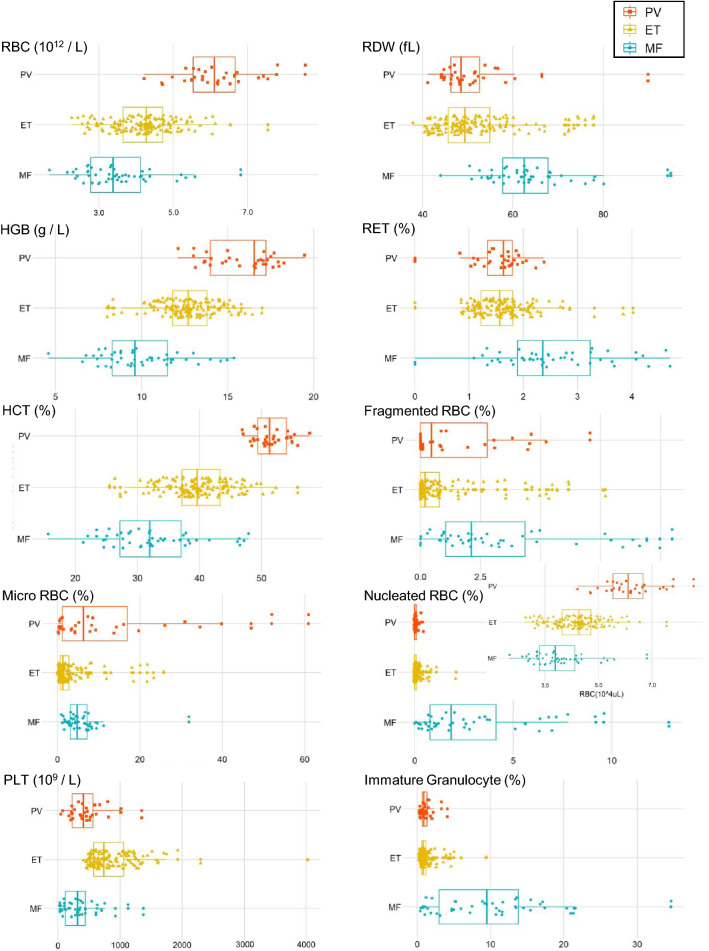
CBC and research parameters in differentiating Ph-negative MPNs extracted from the XN-9000. The box plots **s**how the essential raw data for each case of PV, ET, and MF.

In differentiating Ph-negative MPNs, CBC and research parameters were obtained using the Sysmex XN-9000. Figure 2 shows the essential parameters (raw data) for each category of disease (PV, ET, and MF). In line with WHO diagnostic criteria, PV showed significantly higher values in RBC, hemoglobin (HGB), hematocrit (HCT), and micro RBC than those exhibited by ET and MF; however, the platelet (PLT) count was significantly higher in ET than those of the PV and MF. On the other hand, RBC distribution width (RDW) was the highest in MF, and reticulocytes (RET) and fragmented RBCs were more often seen in MF than the others. Furthermore, immature granulocytes (IGs) and erythroblasts were seen most often in MF. All the XN-9000 parameters obtained from the training data sets were included in the diagnostic analysis of the XGBoost-based DLS, along with the morphological parameters.

### Differentiation of Ph-negative MPNs using deep learning algorithms

**Figure 3:**
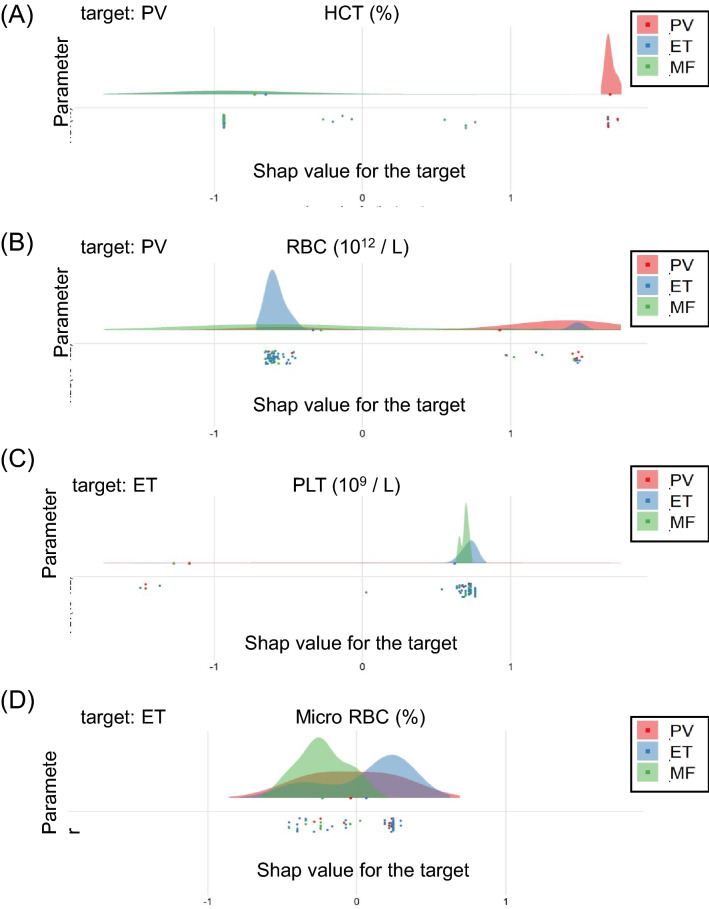

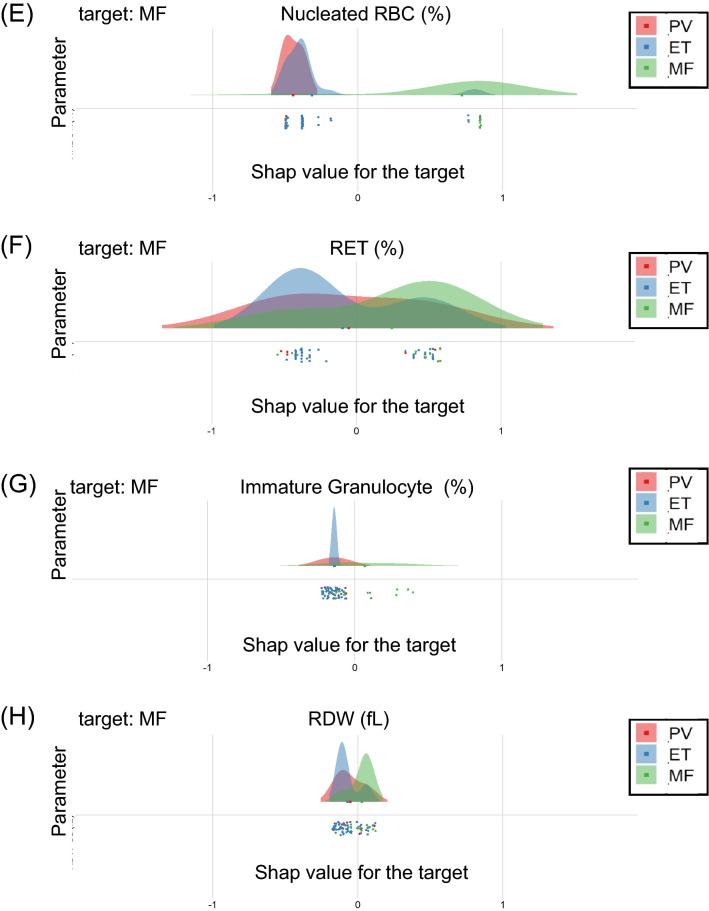
Performance of the DLS-based diagnostic system. (**E**–**H**) The calculated SHAP values are used to outline the useful parameters in the differentiation of Ph-negative MPNs by plotting the SHAP values of positive and negative likelihood to differentiate each disease. SHAP values close to 1 denote high likelihood, whereas those close to -1 denote less likelihood.

The performance of the trained DLS-based diagnostic system was examined using test samples. Figure 3 highlights the useful parameters in the differentiation of Ph-negative MPNs. This was obtained by plotting the SHAP values of the positive and negative likelihood to differentiate each disease. SHAP values close to 1 indicate high likelihood, and values close to -1 imply less likelihood. The SHAP values to differentiate PV from ET and MF showed high probabilities for HCT and RBC (Fig. 3A,B). Although platelet count is an essential diagnostic value for ET, this parameter is also useful to differentiate MF (Fig. 3C). MF and ET was differentiated by micro RBC, nucleated RBC, and RET (Fig. 3D-E). However, the SHAP values for PV overlapped with both ET and MF in micro RBC and RET (Fig. 3D,F), and with ET in nucleated RBC (Fig. 3E). Immature granulocytes and RDW parameters slightly contributed to the differentiation of MF from ET, but not from PV (Fig. 3G,H).

**Figure 4:**
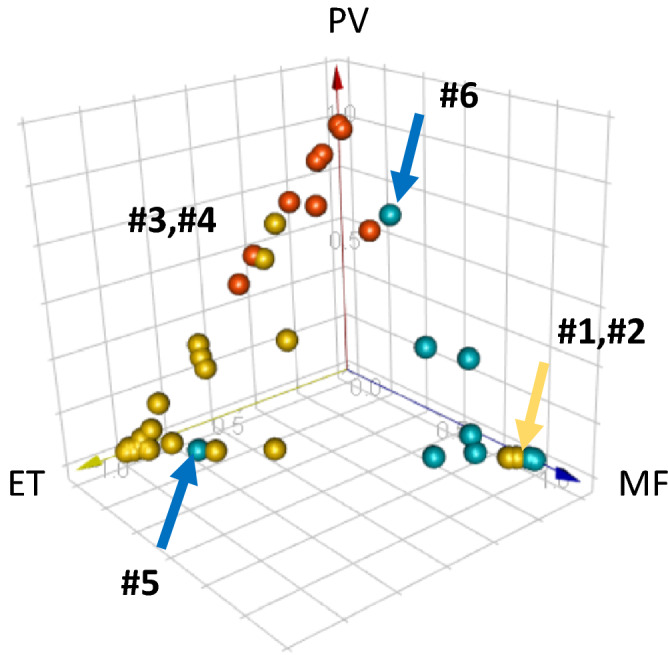
Three-dimensional plot of the predicted likelihood for all cases. Case #1 and Case #2: Definitive diagnosis ET, automated diagnosis MF. Case #3 and Case #4: Definitive diagnosis ET, automated diagnosis PV. Case #5: Definitive diagnosis MF (secondary, post-ET), automated diagnosis ET. Case #6: Definitive diagnosis MF, automated diagnosis PV.

Figure 4 shows a 3-dimensional plot of the predicted probability for all cases. Each case was plotted as a function of the probability of each disease (PV, ET, and MF) on a scale of 0 to 1. Although most of the cases were correctly diagnosed with > 50% confidence, several cases were misclassified by the DLS-based diagnostic system. Two ET cases (yellow arrow, Case #1, #2) with thrombocytosis (PLT: 543 and 659 × 10^9^/L, respectively) were classified as MF possibly due to the presence of immature granulocytes (3.5–4.6%) and nucleated RBCs (0.7%). Two ET cases with elevated hemoglobin (Hb 15.8 g/L, 16.5 g/L) were incorrectly classified as PV despite showing signs of thrombocytosis (PLT > 700 × 10^9^/L) (Case #3, #4). Furthermore, one case of secondary MF (post-ET) was classified as ET (blue arrow, Case #5), and another MF case with immature granulocytes (6%) and nucleated RBC (0.6%) that exhibited elevated hemoglobin was classified as PV (blue arrow, #6).

**Figure 5:**
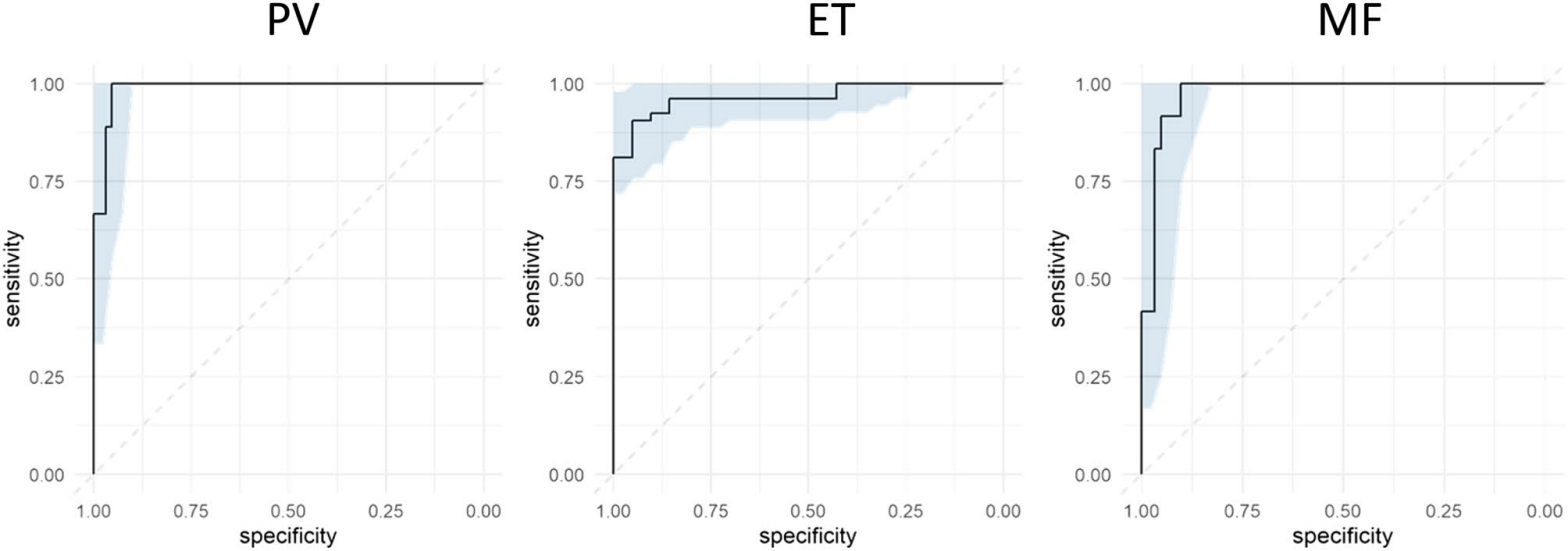
Differential diagnostic performance of the automated diagnostic support system. ROC curves for PV, ET, and MF. The AUC is used to measure performance; the maximum value is 1. Blue shades depict the 95% confidence intervals.

Figure 5 shows the ROC curves of the automated diagnostic support system for the three types of Ph-negative MPNs. The sensitivity and specificity in terms of the differential diagnosis of Ph-negative MPNs were more than 90%, and the calculated AUCs of the ROC curve were 0.990, 0.967, and 0.974 for PV, ET, and MF, respectively (Table 1).

**Table 1:** Performance of the DLS-based diagnostic system in MPN diagnosis.

	Sensitivity (%)	Specificity (%)	AUC
PV	100	95.4	0.988
ET	90.6	95.2	0.967
MF	100	90.3	0.974

## Discussion

In the diagnoses of Ph-negative MPNs, reviews on CBCs and PB smears established the basis of the initial essential workup. Since manual microscopic examinations of PB smears are time-consuming and can cause inaccuracies due to human factors, we developed an automated image diagnostic system using a CNN-based DLS for MDS in 2019^10^. In this study, we sought to further develop a diagnostic support system to differentiate various Ph-negative MPNs, specifically PV, ET, and MF. To this end, we combined the cell count and research parameters obtained from an automated hematology analyzer (XN-9000) with the morphological parameters obtained from the CNN-based image analyses. Subsequently, we utilized the samples of the MF cases with BM fibrosis grades ≥ 2, including PMF, secondary MF, post-PV MF, and post-ET MF. It was demonstrated that the findings of the BM fibrosis for overt PMF and secondary MF with BM fibrosis grade ≥ 2 are almost identical, and their clinical features are similar. Both PMF and MF were capable of causing anemia, while exhibiting a mild change in leukocyte counts and the presence of peripheral erythroblasts and myeloblasts (leukoerythroblastosis), teardrop-shaped erythrocytes, and giant platelets^7^.

Our data were consistent with the features described above since blasts, nucleated RBCs or erythroblasts, and immature leukocytes such as promyelocytes, myelocytes, and metamyelocytes significantly increased in the MF cases as compared to those observed in the EV and PV cases. In our cohort, all the cases misclassified by the DLS-based diagnostic system had multiple characteristic features of different Ph-negative MPNs. For example, one post-ET MF was classified as ET due to thrombocytosis. Another MF case was miss-classified as PV due to its elevated hemoglobin. Masked PV (i.e., early stage PV) can show ET-like thrombocytosis in CBC and PB smear examinations^11,12^. A recent study has shown the reproducibility of BM morphology in masked PV and its differentiation from ET to some extent^12^. In our analyses, two ET cases with elevated hemoglobin as well as thrombocytosis were miss-classified as PV (Fig. 4).

Recently, several research groups have also attempted to develop automated diagnosis systems of hematological disorders. Meggendorfer et al. constructed a deep learning system to differentiate MPNs by combining CBC parameters and genetic information using a NGS panel consisting of 18 genes. The accuracy of this system was 100% in PV, 87% in ET, and 21% in PMF^13^. However, the pathophysiological role of genetic mutations in PMF has not yet been fully understood. For example, approximately 15% of MPN cases were found to be negative for three major driver genes (JAK2, CALR, and MPL)^14^. Gunčar et al.^15^ constructed a machine learning diagnostic system in various hematological diseases. Their random forest algorithms fed with CBC parameters could make relatively accurate diagnoses in up to 88% of hematological disorders such as iron deficiency anemia and lymphoma. However, its accuracy in differentiating malignant diseases was relatively low^15^. Taken these together, it is reasonable to conclude that morphological analyses can be useful to improve the diagnostic accuracy by CNN-based systems. One step further, currently, we are developing an advanced system to incorporate bone marrow analyses and other medical information such as clinical courses and blood chemical findings.

Our work had several limitations. This is a single-center study that uses a small number of cases and only one type of hematology analyzer. In this DLS-based diagnostic system, only CBCs and cellular morphologies in PB smears were included for analyses, and other parts of the workups were not included. For example, serum chemistry such as C-reactive protein (CRP) and lactate dehydrogenase (LDH), and molecular genetic tests performed on *JAK2*, *CALR*, *MPL,* and *CSF3R* were not included due to the following reasons: (1) currently, blood chemistry analyzers and automated hematology analyzers have incompatible interfaces, and hence cannot be connected with each other; (2) molecular genetic tests and molecular cytogenetic tests are performed separately, and cannot be included in the initial diagnostic workup; (3) examinations of BM aspiration and biopsy were not included since automated analyzers of BM samples have not been developed yet. Furthermore, we used samples obtained at only one point in time. Since PV and ET can progress to MF, multiple consecutive samples in each patient need to be evaluated. Currently, we are planning to develop an automated analyzer for BM samples.

In conclusion, the current version of deep learning systems for image analyses still requires a massive database of various cell images. This study is a step toward achieving our ultimate goal of establishing a universal automated diagnosis system for various hematology disorders.

## Materials and methods

### Sample preparation

This study was approved by the Juntendo University Hospital Medical Ethics Committee (Tokyo, Japan). As part of the approval, the ethics committee explicitly waived the need for an informed consent from individual patients since all the samples were de-identified in line with the Declaration of Helsinki. A total of 344 samples were obtained from 234 patients in K_2_EDTA anticoagulant tubes (Terumo, Tokyo, Japan) and processed with the Sysmex XN-9000 (Sysmex Co., Kobe, Japan). The PB smear slides were prepared with May–Grunwald–Giemsa staining using a fully automated slide maker (Sysmex SP-50). The PB smears were scanned using the Sysmex DI-60.

### Image analyses

To perform analyses of the cell images, we employed the CNN-based automatic image-recognition system that we recently developed^10^. Briefly, the deep learning system (DLS) was trained using a total of 695,030 normal and abnormal cell images, and we could accurately classify 17 cell subtypes and detect 97 abnormal morphological features^10^. The neural network was composed of two modules: the first module consisting of series of neural networks layers such as 2 dimensional Separative convolutional neural-network (CNN) based “image extractor” module, and the second module consisting of the layers connected to the output module. Both architectures were implemented by Keras and Tensorflow.

### Analyses using the Sysmex XN-9000

Flow cytometry–based analyses were performed using the automated hematology analyzer, Sysmex XN-9000, (Sysmex Co., Kobe, Japan) to obtain 70 CBC-related reportable and 80 supplemental research parameters. The XN-9000 has three different analytical channels: white cell differential (WDF), white cell nucleated (WNR), and white cell precursor (WPC) channels. The signals from these channels were used to classify the leukocytes into six types, namely: neutrophil, eosinophil, basophil, lymphocyte, monocyte, and immature granulocyte. The fluorescent platelet (PLT-F) channel was used to obtain the parameters of platelet counts, and the reticulocyte (RET) channel was used to obtain the parameters of reticulocytes as described in earlier experiments^16^. The red blood cells were stained almost immediately after lysing, with specific polymethine-based fluorescent dyes that bind to nucleic acid and organelles in the cell. Three fluorescence signals were then obtained using a 633-nm laser light: forward scatter (FSC), side scatter (SSC), and side fluorescence (SFL). Two-dimensional scattergrams were automatically analyzed using these signals to determine cell size, cytoplasmic complexity, and RNA and DNA amounts.

### Training and analyses of the combined automated diagnostic system

For the training data sets, we utilized cell images scanned by the DI-60, along with the CBC and the research parameters of the XN-9000 obtained via the polycythemia vera (PV, n = 23), essential thrombocytosis (ET, n = 101), and myelofibrosis (MF, n = 36) cases. The MF cases included primary- and secondary-MF (post-PV and post-ET) with BM fibrosis classified as grade 2 or 3 through BM biopsies (based on the European consensus on the grading of BM fibrosis)^17^. All diagnoses were confirmed by independent hematopathologists based on clinical information, laboratory data, flow cytometry analyses, genetic data, BM aspiration, and biopsy findings according to the WHO criteria^5^. The DLS was trained using a total of 174 parameters obtained from the XN-9000, and 114 cell morphology parameters obtained from the CNN-based image-recognition system. The XN parameters comprised 70 CBC parameters, 80 research parameters, and 24 flag message parameters, whereas the morphology parameters comprised 17 parameters to classify different cell types and 97 parameters to recognize abnormal dysmorphic features. Table S1 summarize these parameters. The diagnostic-aid algorithm to differentiate PV, ET and MF was trained using XGBoost algorithm that was fed with the cell-image derived DLS parameters (17 + 97) and the XN-derived parameters. Each of the parameter used here was evaluated using the XGBoost algorithm based on the SHAP value of dysmorphic extents in various cell types^18^. We tested our system using samples obtained from nine PV, 53 ET, and 12 MF patients.

### Statistics

Data were analyzed for statistical significance using R 3.6.2 with the stats package. Analysis of variance (ANOVA) was used to calculate the significance in more than three groups. Any case with *p* ≤ 0.05 was considered statistically significant. The area under the curve (AUC) of the receiver operating characteristic (ROC) curve was calculated using the pROC package in R^19^.

## Supplementary Information

Supplementary Information.

## Data Availability

The datasets generated during and/or analyzed during the current study are available from the corresponding author on reasonable request.
