# Supplementary material for: Automated diagnostic support system with deep learning algorithms for distinction of Philadelphia chromosome-negative myeloproliferative neoplasms using peripheral blood specimen
Source: Sci Rep. 2021 Feb 9;11:3367. doi: 10.1038/s41598-021-82826-9 (PMC7873208; doi:10.1038/s41598-021-82826-9)
Supplement: Supplementary file 1 — Supplementary Information. [file 41598_2021_82826_MOESM1_ESM.docx]

**Title:**

**Automated diagnostic support system with deep learning algorithms for distinction of Philadelphia chromosome-negative myeloproliferative neoplasms using peripheral blood specimen**

**Authors/Affiliations**

Konobu Kimura^1^, Tomohiko Ai^2^, Yuki Horiuchi^2^, Akihiko Matsuzaki^1^, Kumiko Nishibe^1^, Setsuko Marutani^1^, Kaori Saito^1,2^, Kimiko Kaniyu^1^, Ikki Takehara^3^, Kinya Uchihashi^3^, Akimichi Ohsaka^1^, *Yoko Tabe^1,^^2^

^1^Department of Next Generation Hematology Laboratory Medicine, Juntendo University Graduate School of Medicine, Tokyo, Japan.

^2^Department of Clinical Laboratory Medicine, Juntendo University Graduate School of Medicine, Tokyo, Japan.

^3^Sysmex, Kobe, Japan

**Corresponding Author:** Yoko Tabe, M.D. Ph.D., Department of Next Generation Hematology Laboratory Medicine, Juntendo University Graduate School of Medicine 2-1-1, Hongo, Bunkyo-ku, Tokyo 113-8421 JAPAN

**SUPPROTING INFORMATION**

**Supplementary Table S1. Parameters used for xgboost algorithm**

| Parameter name | explanation | Category | Parameter attribute |
| --- | --- | --- | --- |
| WBC | White blood cell (leukocyte) count | CBC(XN) | reportable |
| RBC | Red blood cell (erythrocyte) count | CBC(XN) | reportable |
| HGB | Hemoglobin concentration | CBC(XN) | reportable |
| HCT | Hematocrit | CBC(XN) | reportable |
| MCV | Mean corpuscular volume | CBC(XN) | reportable |
| MCH | Mean corpuscular hemoglobin | CBC(XN) | reportable |
| MCHC | Mean corpuscular hemoglobin concentration | CBC(XN) | reportable |
| PLT | Platelet count | CBC(XN) | reportable |
| RDW-SD | Red cell distribution width (standard deviation) | CBC(XN) | reportable |
| RDW-CV | Red cell distribution width (coefficient of variation) | CBC(XN) | reportable |
| PDW | Platelet distribution width | CBC(XN) | reportable |
| MPV | Mean platelet volume | CBC(XN) | reportable |
| P-LCR | Platelet-large cell ratio | CBC(XN) | reportable |
| PCT | Plateletcrit | CBC(XN) | reportable |
| NRBC# | Nucleated red blood cell count | CBC(XN) | reportable |
| NRBC% | Nucleated red blood cell percent | CBC(XN) | reportable |
| NEUT# | Neutrophil count | CBC(XN) | reportable |
| LYMPH# | Lymphocyte count | CBC(XN) | reportable |
| MONO# | Monocyte count | CBC(XN) | reportable |
| EO# | Eosinophil count | CBC(XN) | reportable |
| BASO# | Basophil count | CBC(XN) | reportable |
| IG# | Immature granulocyte count | CBC(XN) | reportable |
| NEUT% | Neutrophil percent | CBC(XN) | reportable |
| LYMPH% | Lymphocyte percent | CBC(XN) | reportable |
| MONO% | Monocyte percent | CBC(XN) | reportable |
| EO% | Eosinophil percent | CBC(XN) | reportable |
| BASO% | Basophil percent | CBC(XN) | reportable |
| IG% | Immature granulocyte percent | CBC(XN) | reportable |
| RET% | Reticulocyte percent | CBC(XN) | reportable |
| RET# | Reticulocyte count | CBC(XN) | reportable |
| IRF | Immature reticulocyte fraction | CBC(XN) | reportable |
| LFR | Low fluorescence ratio | CBC(XN) | reportable |
| MFR | Medium fluorescence ratio | CBC(XN) | reportable |
| RET-He | High fluorescence ratio | CBC(XN) | reportable |
| IPF | Immature platelet fraction | CBC(XN) | reportable |
| WBC/M | The Notation for WBC indicating the parameter is abnormal | CBC(XN) | reportable |
| RBC/M | The Notation for RBC indicating the parameter is abnormal | CBC(XN) | reportable |
| HGB/M | The Notation for HGB indicating the parameter is abnormal | CBC(XN) | reportable |
| HCT/M | The Notation for HCT indicating the parameter is abnormal | CBC(XN) | reportable |
| MCV/M | The Notation for MCV indicating the parameter is abnormal | CBC(XN) | reportable |
| MCH/M | The Notation for MCH indicating the parameter is abnormal | CBC(XN) | reportable |
| MCHC/M | The Notation for MCHC indicating the parameter is abnormal | CBC(XN) | reportable |
| PLT/M | The Notation for PLT indicating the parameter is abnormal | CBC(XN) | reportable |
| RDW-SD/M | The Notation for RDW-SD indicating the parameter is abnormal | CBC(XN) | reportable |
| RDW-CV/M | The Notation for RDW-CV indicating the parameter is abnormal | CBC(XN) | reportable |
| PDW/M | The Notation for PDW indicating the parameter is abnormal | CBC(XN) | reportable |
| MPV/M | The Notation for MPV indicating the parameter is abnormal | CBC(XN) | reportable |
| P-LCR/M | The Notation for P-LCR indicating the parameter is abnormal | CBC(XN) | reportable |
| PCT/M | The Notation for PCT indicating the parameter is abnormal | CBC(XN) | reportable |
| NRBC#/M | The Notation for NRBC# indicating the parameter is abnormal | CBC(XN) | reportable |
| NRBC%/M | The Notation for NRBC% indicating the parameter is abnormal | CBC(XN) | reportable |
| NEUT#/M | The Notation for NEUT# indicating the parameter is abnormal | CBC(XN) | reportable |
| LYMPH#/M | The Notation for LYMPH# indicating the parameter is abnormal | CBC(XN) | reportable |
| MONO#/M | The Notation for MONO# indicating the parameter is abnormal | CBC(XN) | reportable |
| EO#/M | The Notation for EO# indicating the parameter is abnormal | CBC(XN) | reportable |
| BASO#/M | The Notation for BASO# indicating the parameter is abnormal | CBC(XN) | reportable |
| IG#/M | The Notation for IG# indicating the parameter is abnormal | CBC(XN) | reportable |
| NEUT%/M | The Notation for NEUT% indicating the parameter is abnormal | CBC(XN) | reportable |
| LYMPH%/M | The Notation for LYMPH% indicating the parameter is abnormal | CBC(XN) | reportable |
| MONO%/M | The Notation for MONO% indicating the parameter is abnormal | CBC(XN) | reportable |
| EO%/M | The Notation for EO% indicating the parameter is abnormal | CBC(XN) | reportable |
| BASO%/M | The Notation for BASO% indicating the parameter is abnormal | CBC(XN) | reportable |
| IG%/M | The Notation for IG% indicating the parameter is abnormal | CBC(XN) | reportable |
| RET%/M | The Notation for RET% indicating the parameter is abnormal | CBC(XN) | reportable |
| RET#/M | The Notation for RET# indicating the parameter is abnormal | CBC(XN) | reportable |
| IRF/M | The Notation for IRF indicating the parameter is abnormal | CBC(XN) | reportable |
| LFR/M | The Notation for LFR indicating the parameter is abnormal | CBC(XN) | reportable |
| MFR/M | The Notation for MFR indicating the parameter is abnormal | CBC(XN) | reportable |
| RET-He/M | The Notation for RET-He indicating the parameter is abnormal | CBC(XN) | reportable |
| IPF/M | The Notation for IPF indicating the parameter is abnormal | CBC(XN) | reportable |
|  |  |  |  |

| Parameter name | explanation | category | Parameter attribute |
| --- | --- | --- | --- |
| TNC | The total nuclear cell count (WBC#+NRBC#) | CBC(XN) | Research |
| WBC-N | The WBC count calculated from the WNR channel | CBC(XN) | Research |
| TNC-N | The total nuclear cell count (WBC#+NRBC#) calculated from the WNR channel | CBC(XN) | Research |
| BA-N# | The basophil counts calculated from the WNR channel | CBC(XN) | Research |
| BA-N% | The basophil percent calculated from the WNR channel | CBC(XN) | Research |
| WBC-D | The WBC count calculated from the WDF channel | CBC(XN) | Research |
| TNC-D | The total nuclear cell count (WBC#+NRBC#) calculated from the WDF channel | CBC(XN) | Research |
| NEUT#& | The number of particles obtained by subtracting the IG count from the NEUT count | CBC(XN) | Research |
| NEUT%& | The ratio of the count obtained by subtracting IG# from NEUT# to the WBC count | CBC(XN) | Research |
| LYMP#& | The number of particles obtained by subtracting the HFLC count from the LYMPH count | CBC(XN) | Research |
| LYMP%& | The ratio of the count obtained by subtracting HFLC# from LYMPH# to the WBC count | CBC(XN) | Research |
| HFLC# | The count of the upper LYMPH area of the WDF scattergram | CBC(XN) | Research |
| HFLC% | The ratio of the count of the upper LYMPH area of the WDF scattergram to the WBC count | CBC(XN) | Research |
| BA-D# | The basophil counts calculated from the WDF channel | CBC(XN) | Research |
| BA-D% | The basophil percent calculated from the WDF channel | CBC(XN) | Research |
| NE-SSC | The lateral scattered light intensity of the NEUT area on the WDF scattergram | CBC(XN) | Research |
| NE-SFL | The fluorescent light intensity of the NEUT area on the WDF scattergram | CBC(XN) | Research |
| NE-FSC | The forward-scattered light intensity of the NEUT area on the WDF scattergram | CBC(XN) | Research |
| LY-X | The lateral scattered light intensity of the LYMPH area on the WDF scattergram | CBC(XN) | Research |
| LY-Y | The fluorescent light intensity of the LYMPH area on the WDF scattergram | CBC(XN) | Research |
| LY-Z | The forward-scattered light intensity of the LYMPH area on the WDF scattergram | CBC(XN) | Research |
| MO-X | The lateral scattered light intensity of the MONO area on the WDF scattergram | CBC(XN) | Research |
| MO-Y | The fluorescent light intensity of the MONO area on the WDF scattergram | CBC(XN) | Research |
| MO-Z | The forward-scattered light intensity of the MONO area on the WDF scattergram | CBC(XN) | Research |
| NE-WX | The lateral scattered light distribution width index of the NEUT area on the WDF scattergram | CBC(XN) | Research |
| NE-WY | The fluorescent light distribution width index of the NEUT area on the WDF scattergram | CBC(XN) | Research |
| NE-WZ | The forward-scattered light distribution width index of the NEUT area on the WDF scattergram | CBC(XN) | Research |
| LY-WX | The lateral scattered light distribution width index of the LYMPH area on the WDF scattergram | CBC(XN) | Research |
| LY-WY | LY-WY The fluorescent light distribution width index of the LYMPH area on the WDF scattergram | CBC(XN) | Research |
| LY-WZ | The forward-scattered light distribution width index of the LYMPH area on the WDF scattergram | CBC(XN) | Research |
| MO-WX | The lateral scattered light distribution width index of the MONO area on the WDF scattergram | CBC(XN) | Research |
| MO-WY | The fluorescent light distribution width index of the MONO area on the WDF scattergram | CBC(XN) | Research |
| MO-WZ | The forward-scattered light distribution width index of the MONO area on the WDF scattergram | CBC(XN) | Research |
| WBC-P | WBC count calculated from WPC channel | CBC(XN) | Research |
| TNC-P | The total nuclear cell count(WBC#+NRBC#) calculated from WPC channel | CBC(XN) | Research |
| PLT-I | The PLT count calculated from the RBC/PLT channel (PLT distributions) | CBC(XN) | Research |
| RBC-He | Reticulocyte hemoglobin equivalent | CBC(XN) | Research |
| MicroR | Micro RBC ratio | CBC(XN) | Research |
| MacroR | Macro RBC ratio | CBC(XN) | Research |
| HYPO-He | The ratio of the count in the low level area of the forward scattered light signal in the RBC (mature red blood cell) area of the RET scattergram, to mature red blood cells | CBC(XN) | Research |
| HYPER-He | The ratio of the count in the high level area of the forward scattered light signal in the RBC (mature red blood cell) area of the RET scattergram, to mature red blood cells | CBC(XN) | Research |
| RBC-O | RBC count calculated from the RET channel | CBC(XN) | Research |
| PLT-O | PLT count calculated from the RET channel | CBC(XN) | Research |
| RET-Y | The forward scattered light intensity of the RET area on the RET scattergram | CBC(XN) | Research |
| RET-RBC-Y | The forward scattered light intensity of RBC (mature red blood cells) area on the RET | CBC(XN) | Research |
| IRF-Y | scattergram | CBC(XN) | Research |
| FRC# | The intensity of forward scattered light from the IRF area on the RET scattergram | CBC(XN) | Research |
| FRC% | The absolute count calculated from the count in a specific area below the RBC area in the | CBC(XN) | Research |
| RPI | RET scattergram | CBC(XN) | Research |
| RET-UPP | The ratio calculated from the count in a specific area below the RBC area in the RET scattergram | CBC(XN) | Research |
| RET-TNC | Reticulocyte Production Index | CBC(XN) | Research |
| HGB-O | The count in the UPP area of the RET scattergram | CBC(XN) | Research |
| MCHC-O | The count in the TNC area of the RET scattergram | CBC(XN) | Research |
| Delta-HGB | Hemoglobin concentration calculated from the RET channel | CBC(XN) | Research |
| PLT-F | MCHC-O is calculated by the equation HGB-O / HCT | CBC(XN) | Research |
| H-IPF | Delta-HGB is calculated by the equation HGB - HGB-O | CBC(XN) | Research |
| PLT-F2 | The platelet count calculated from the PLT-F channel | CBC(XN) | Research |
| IPF# |  | CBC(XN) | Research |
| AS-LYMP# | Count of activated lymphocytes (plasma cells) reflecting the highest fluorescence signals in RE-LYMP cell population | CBC(XN) | Research |
| AS-LYMP% | Percentage of activated lymphocytes (plasma cells) reflecting the highest fluorescence signals in RE-LYMP cell population | CBC(XN) | Research |
| AS-LYMP%L | The ratio of the AS-LYMP count to the lymphocyte count | CBC(XN) | Research |
| RE-LYMP%L | The ratio of the RE-LYMP count to the lymphocyte count | CBC(XN) | Research |
| RE-LYMP# | Count of reactive lymphocytes defined as all lymphocytes reflecting higher fluorescence signals than normal lymphocytes | CBC(XN) | Research |
| RE-LYMP% | Percentage of lymphocyte reactive lymphocytes defined as all lymphocytes reflecting higher fluorescence signals than normal lymphocytes | CBC(XN) | Research |
| NEUT-RI | Neutrophil reactivity intensity | CBC(XN) | Research |
| NEUT-GI | Neutrophil granularity intensity | CBC(XN) | Research |
| WBC(hsA) | WBC counted in the WDF channel using hsA mode | CBC(XN) | Research |
| RBC-I(hsA) | RBC counted in the RBC/PLT channel (RBC distribution) using hsA mode | CBC(XN) | Research |
| RBC-O(hsA) | RBC counted in the RET channel using hsA mode | CBC(XN) | Research |
| NEUT#(hsA) | The count in the NEUT area of the WDF scattergram using hsA mode | CBC(XN) | Research |
| LYMP#(hsA) | The count in the LYMPH area of the WDF scattergram using hsA mode | CBC(XN) | Research |
| MONO#(hsA) | The count in the MONO area of the WDF scattergram using hsA mode | CBC(XN) | Research |
| EO#(hsA) | The count appearing in the EO area of the WDF scattergram using hsA mode | CBC(XN) | Research |
| NEUT%(hsA) | NEUT count divided by WBC and expressed as a percentage using hsA mode | CBC(XN) | Research |
| LYMP%(hsA) | LYMPH count divided by WBC and expressed as a percentage using hsA mode | CBC(XN) | Research |
| MONO%(hsA) | MONO count divided by WBC and expressed as a percentage using hsA mode | CBC(XN) | Research |
| EO%(hsA) | EO count divided by WBC and expressed as a percentage using hsA mode | CBC(XN) | Research |
| HF#(hsA) | The count in the area with stronger fluorescence than the WBC area of the WDF | CBC(XN) | Research |
| HF%(hsA) | scattergram using hsA mode | CBC(XN) | Research |
| TC#(hsA) | HF count divided by WBC and expressed as a percentage using hsA mode | CBC(XN) | Research |
|  |  |  |  |

| Parameter name | explanation | category | Parameter attribute |
| --- | --- | --- | --- |
| WBC Abn Scattergram | Abnormal WBC scattergram | CBC(XN) | Flag message |
| NRBC Present | High nucleated RBC count | CBC(XN) | Flag message |
| IG Present | Increased immature granulocyte | CBC(XN) | Flag message |
| Blasts? | Possibility that blasts are present | CBC(XN) | Flag message |
| Abn Lympho? | Possibility of abnormal lymphocytes | CBC(XN) | Flag message |
| Left Shift? | Possibility of left shift | CBC(XN) | Flag message |
| Atypical Lympho? | Possibility of atypical lymphocytes | CBC(XN) | Flag message |
| RBC Abn Distribution | Abnormal RBC distribution | CBC(XN) | Flag message |
| Dimorphic Population | Double-peak RBC distribution | CBC(XN) | Flag message |
| RET Abn Scattergram | Abnormal RET scattergram | CBC(XN) | Flag message |
| Anisocytosis | Anisocytosis | CBC(XN) | Flag message |
| Microcytosis | Microcytosis | CBC(XN) | Flag message |
| Macrocytosis | Macrocytosis | CBC(XN) | Flag message |
| Hypochromia | Hypochromia | CBC(XN) | Flag message |
| Anemia | Anemia | CBC(XN) | Flag message |
| RBC Agglutination? | Possibility of RBC agglutination | CBC(XN) | Flag message |
| Turbidity/HGB Interf? | Possibility of effect on HGB by chylemia | CBC(XN) | Flag message |
| Iron Deficiency? | Possibility of iron deficiency | CBC(XN) | Flag message |
| HGB Defect? | Possibility of HGB abnormality | CBC(XN) | Flag message |
| Fragments? | Possibility of fragmented red blood cells | CBC(XN) | Flag message |
| PLT Abn Distribution | Abnormal PLT distribution | CBC(XN) | Flag message |
| PLT Abn Scattergram | Abnormal PLT scattergram | CBC(XN) | Flag message |
| PLT Clumps? | Possibility of PLT clumps | CBC(XN) | Flag message |
| Giant Platelet? | Giant platelets | CBC(XN) | Flag message |
|  |  |  |  |

| Parameter name | explanation | Category | Parameter attribute |
| --- | --- | --- | --- |
| Segmented Neutrophil | Segmented Neutrophil percentage | Morphology by DLS | Cell type |
| Band Neutrophil | Band Neutrophil percentage | Morphology by DLS | Cell type |
| Metamyelocyte | Metamyelocyte percentage | Morphology by DLS | Cell type |
| Myelocyte | Myelocyte percentage | Morphology by DLS | Cell type |
| Promyelocyte | Promyelocyte percentage | Morphology by DLS | Cell type |
| Blast | Blast percentage | Morphology by DLS | Cell type |
| Lymphocyte | Lymphocyte percentage | Morphology by DLS | Cell type |
| Variant Lymphocyte | Variant Lymphocyte percentage | Morphology by DLS | Cell type |
| Monocyte | Monocyte percentage | Morphology by DLS | Cell type |
| Eosinophil | Eosinophil percentage | Morphology by DLS | Cell type |
| Basophil | Basophil percentage | Morphology by DLS | Cell type |
| Large Platelet | Large Platelet percentage | Morphology by DLS | Cell type |
| Megakaryocyte | Megakaryocyte percentage | Morphology by DLS | Cell type |
| Platelet Aggregation | Platelet Aggregation percentage | Morphology by DLS | Cell type |
| Erythroblast | Erythroblast percentage | Morphology by DLS | Cell type |
| Smudge | Smudge percentage | Morphology by DLS | Cell type |
| Artifact | Artifact percentage | Morphology by DLS | Cell type |
|  |  |  |  |

| Parameter name | explanation | Category | Parameter attribute |
| --- | --- | --- | --- |
| Blast :  Auer rods | Cell percentage having the morphology feature of Blast and Auer rods | Morphology by DLS | Abnormal feature |
| Blast :  abnormal shape of nuclei | Cell percentage having the morphology feature of Blast and abnormal shape of nuclei | Morphology by DLS | Abnormal feature |
| Blast :  abnormal granulation | Cell percentage having the morphology feature of Blast and abnormal granulation | Morphology by DLS | Abnormal feature |
| Blast :  vacuoles | Cell percentage having the morphology feature of Blast and vacuoles | Morphology by DLS | Abnormal feature |
| Blast :  smudge | Cell percentage having the morphology feature of Blast and smudge | Morphology by DLS | Abnormal feature |
| Blast :  apoptotic cell | Cell percentage having the morphology feature of Blast and apoptotic cell | Morphology by DLS | Abnormal feature |
| Promyelocyte :  Auer rods | Cell percentage having the morphology feature of Promyelocyte and Auer rods | Morphology by DLS | Abnormal feature |
| Promyelocyte :  fagott cell | Cell percentage having the morphology feature of Promyelocyte and fagott cell | Morphology by DLS | Abnormal feature |
| Promyelocyte :  abnormal shape of nuclei | Cell percentage having the morphology feature of Promyelocyte and abnormal shape of nuclei | Morphology by DLS | Abnormal feature |
| Promyelocyte :  abnormal granulation | Cell percentage having the morphology feature of Promyelocyte and abnormal granulation | Morphology by DLS | Abnormal feature |
| Promyelocyte :  degranulation | Cell percentage having the morphology feature of Promyelocyte and degranulation | Morphology by DLS | Abnormal feature |
| Promyelocyte :  vacuoles | Cell percentage having the morphology feature of Promyelocyte and vacuoles | Morphology by DLS | Abnormal feature |
| Promyelocyte :  smudge | Cell percentage having the morphology feature of Promyelocyte and smudge | Morphology by DLS | Abnormal feature |
| Promyelocyte :  apoptotic cell | Cell percentage having the morphology feature of Promyelocyte and apoptotic cell | Morphology by DLS | Abnormal feature |
| Myelocyte :  Auer rods | Cell percentage having the morphology feature of Myelocyte and Auer rods | Morphology by DLS | Abnormal feature |
| Myelocyte :  abnormal shape of nuclei | Cell percentage having the morphology feature of Myelocyte and abnormal shape of nuclei | Morphology by DLS | Abnormal feature |
| Myelocyte :  abnormal granulation | Cell percentage having the morphology feature of Myelocyte and abnormal granulation | Morphology by DLS | Abnormal feature |
| Myelocyte :  degranulation | Cell percentage having the morphology feature of Myelocyte and degranulation | Morphology by DLS | Abnormal feature |
| Myelocyte :  vacuoles | Cell percentage having the morphology feature of Myelocyte and vacuoles | Morphology by DLS | Abnormal feature |
| Myelocyte :  smudge | Cell percentage having the morphology feature of Myelocyte and smudge | Morphology by DLS | Abnormal feature |
| Myelocyte :  apoptotic cell | Cell percentage having the morphology feature of Myelocyte and apoptotic cell | Morphology by DLS | Abnormal feature |
| Metamyelocyte :  Auer rods | Cell percentage having the morphology feature of Metamyelocyte and Auer rods | Morphology by DLS | Abnormal feature |
| Metamyelocyte :  abnormal shape of nuclei | Cell percentage having the morphology feature of Metamyelocyte and abnormal shape of nuclei | Morphology by DLS | Abnormal feature |
| Metamyelocyte :  abnormal granulation | Cell percentage having the morphology feature of Metamyelocyte and abnormal granulation | Morphology by DLS | Abnormal feature |
| Metamyelocyte :  degranulation | Cell percentage having the morphology feature of Metamyelocyte and degranulation | Morphology by DLS | Abnormal feature |
| Metamyelocyte :  vacuoles | Cell percentage having the morphology feature of Metamyelocyte and vacuoles | Morphology by DLS | Abnormal feature |
| Metamyelocyte :  giant | Cell percentage having the morphology feature of Metamyelocyte and giant | Morphology by DLS | Abnormal feature |
| Metamyelocyte :  smudge | Cell percentage having the morphology feature of Metamyelocyte and smudge | Morphology by DLS | Abnormal feature |
| Metamyelocyte :  apoptotic cell | Cell percentage having the morphology feature of Metamyelocyte and apoptotic cell | Morphology by DLS | Abnormal feature |
| Granulocyte :  Auer rods | Cell percentage having the morphology feature of Granulocyte and Auer rods | Morphology by DLS | Abnormal feature |
| Granulocyte :  Pelger-Huet anomaly | Cell percentage having the morphology feature of Granulocyte and Pelger-Huet anomaly | Morphology by DLS | Abnormal feature |
| Granulocyte :  spherical /ovoid nucleus | Cell percentage having the morphology feature of Granulocyte and spherical /ovoid nucleus | Morphology by DLS | Abnormal feature |
| Granulocyte :  ring-shaped nuclei | Cell percentage having the morphology feature of Granulocyte and ring-shaped nuclei | Morphology by DLS | Abnormal feature |
| Granulocyte :  hypersegmentation | Cell percentage having the morphology feature of Granulocyte and hypersegmentation | Morphology by DLS | Abnormal feature |
| Granulocyte :  abnormal shape of nuclei | Cell percentage having the morphology feature of Granulocyte and abnormal shape of nuclei | Morphology by DLS | Abnormal feature |
| Granulocyte :  giant | Cell percentage having the morphology feature of Granulocyte and giant | Morphology by DLS | Abnormal feature |
| Granulocyte :  degranulation | Cell percentage having the morphology feature of Granulocyte and degranulation | Morphology by DLS | Abnormal feature |
| Granulocyte :  abnormal granulation | Cell percentage having the morphology feature of Granulocyte and abnormal granulation | Morphology by DLS | Abnormal feature |
| Granulocyte :  toxic granulations | Cell percentage having the morphology feature of Granulocyte and toxic granulations | Morphology by DLS | Abnormal feature |
| Granulocyte :  Döhle body | Cell percentage having the morphology feature of Granulocyte and Döhle body | Morphology by DLS | Abnormal feature |
| Granulocyte :  vacuoles | Cell percentage having the morphology feature of Granulocyte and vacuoles | Morphology by DLS | Abnormal feature |
| Granulocyte :  smudge | Cell percentage having the morphology feature of Granulocyte and smudge | Morphology by DLS | Abnormal feature |
| Granulocyte :  apoptotic cell | Cell percentage having the morphology feature of Granulocyte and apoptotic cell | Morphology by DLS | Abnormal feature |
| Granulocyte :  platelet satellitism | Cell percentage having the morphology feature of Granulocyte and platelet satellitism | Morphology by DLS | Abnormal feature |
| Eosinophil :  abnormal shape of nuclei | Cell percentage having the morphology feature of Eosinophil and abnormal shape of nuclei | Morphology by DLS | Abnormal feature |
| Eosinophil :  abnormal granulation | Cell percentage having the morphology feature of Eosinophil and abnormal granulation | Morphology by DLS | Abnormal feature |
| Eosinophil :  abnormal granulation | Cell percentage having the morphology feature of Eosinophil and abnormal granulation | Morphology by DLS | Abnormal feature |
| Eosinophil :  immature eosinophil | Cell percentage having the morphology feature of Eosinophil and immature eosinophil | Morphology by DLS | Abnormal feature |
| Eosinophil :  vacuoles | Cell percentage having the morphology feature of Eosinophil and vacuoles | Morphology by DLS | Abnormal feature |
| Eosinophil :  smudge | Cell percentage having the morphology feature of Eosinophil and smudge | Morphology by DLS | Abnormal feature |
| Eosinophil :  apoptotic cell | Cell percentage having the morphology feature of Eosinophil and apoptotic cell | Morphology by DLS | Abnormal feature |
| Basophil :  abnormal shape of nuclei | Cell percentage having the morphology feature of Basophil and abnormal shape of nuclei | Morphology by DLS | Abnormal feature |
| Basophil :  abnormal granulation | Cell percentage having the morphology feature of Basophil and abnormal granulation | Morphology by DLS | Abnormal feature |
| Basophil :  abnormal granulation | Cell percentage having the morphology feature of Basophil and abnormal granulation | Morphology by DLS | Abnormal feature |
| Basophil :  immature basophil | Cell percentage having the morphology feature of Basophil and immature basophil | Morphology by DLS | Abnormal feature |
| Basophil :  smudge | Cell percentage having the morphology feature of Basophil and smudge | Morphology by DLS | Abnormal feature |
| Basophil :  apoptotic cell | Cell percentage having the morphology feature of Basophil and apoptotic cell | Morphology by DLS | Abnormal feature |
| Lymphocyte :  plasma cell like | Cell percentage having the morphology feature of Lymphocyte and plasma cell like | Morphology by DLS | Abnormal feature |
| Lymphocyte :  cleaved nuclei | Cell percentage having the morphology feature of Lymphocyte and cleaved nuclei | Morphology by DLS | Abnormal feature |
| Lymphocyte :  flower cell | Cell percentage having the morphology feature of Lymphocyte and flower cell | Morphology by DLS | Abnormal feature |
| Lymphocyte :  multiple nuclei | Cell percentage having the morphology feature of Lymphocyte and multiple nuclei | Morphology by DLS | Abnormal feature |
| Lymphocyte :  increased N:C ratio | Cell percentage having the morphology feature of Lymphocyte and increased N:C ratio | Morphology by DLS | Abnormal feature |
| Lymphocyte :  nucleoloid bodies | Cell percentage having the morphology feature of Lymphocyte and nucleoloid bodies | Morphology by DLS | Abnormal feature |
| Lymphocyte:  abnormal nucleoreticulum | Cell percentage having the morphology feature of Lymphocyte and abnormal nucleoreticulum | Morphology by DLS | Abnormal feature |
| Lymphocyte :  abnormal shape of nuclei | Cell percentage having the morphology feature of Lymphocyte and abnormal shape of nuclei | Morphology by DLS | Abnormal feature |
| Lymphocyte :  Hairy cell like | Cell percentage having the morphology feature of Lymphocyte and Hairy cell like | Morphology by DLS | Abnormal feature |
| Lymphocyte :  bleb like | Cell percentage having the morphology feature of Lymphocyte and bleb like | Morphology by DLS | Abnormal feature |
| Lymphocyte :  granular lymphocyte | Cell percentage having the morphology feature of Lymphocyte and granular lymphocyte | Morphology by DLS | Abnormal feature |
| Lymphocyte :  vacuoles | Cell percentage having the morphology feature of Lymphocyte and vacuoles | Morphology by DLS | Abnormal feature |
| Lymphocyte :  smudge | Cell percentage having the morphology feature of Lymphocyte and smudge | Morphology by DLS | Abnormal feature |
| Lymphocyte :  apoptotic cell | Cell percentage having the morphology feature of Lymphocyte and apoptotic cell | Morphology by DLS | Abnormal feature |
| Variant Lymphocyte :  granular lymphocyte | Cell percentage having the morphology feature of Variant Lymphocyte and granular lymphocyte | Morphology by DLS | Abnormal feature |
| Variant Lymphocyte :  vacuoles | Cell percentage having the morphology feature of Variant Lymphocyte and vacuoles | Morphology by DLS | Abnormal feature |
| Variant Lymphocyte :  smudge | Cell percentage having the morphology feature of Variant Lymphocyte and smudge | Morphology by DLS | Abnormal feature |
| Variant Lymphocyte :  apoptotic cell | Cell percentage having the morphology feature of Variant Lymphocyte and apoptotic cell | Morphology by DLS | Abnormal feature |
| Variant Lymphocyte :  nucleoloid bodies | Cell percentage having the morphology feature of Variant Lymphocyte and nucleoloid bodies | Morphology by DLS | Abnormal feature |
| Variant Lymphocyte :  abnormal shape of nuclei | Cell percentage having the morphology feature of Variant Lymphocyte and abnormal shape of nuclei | Morphology by DLS | Abnormal feature |
| Monocyte :  abnormal shape of nuclei | Cell percentage having the morphology feature of Monocyte and abnormal shape of nuclei | Morphology by DLS | Abnormal feature |
| Monocyte :  abnormal granulation | Cell percentage having the morphology feature of Monocyte and abnormal granulation | Morphology by DLS | Abnormal feature |
| Monocyte :  smudge | Cell percentage having the morphology feature of Monocyte and smudge | Morphology by DLS | Abnormal feature |
|  |  |  |  |
